# Navigating spatial barriers in healthcare: health equity and end-of-life care infrastructure in South Korea and China

**DOI:** 10.3389/fpubh.2026.1827548

**Published:** 2026-07-13

**Authors:** Songwu Luo, Yanguang Yun, Dongje Cho, Jaehyun Cho

**Affiliations:** Department of Law, Dong-A University, Busan, Republic of Korea

**Keywords:** China, comparative health policy, geographic accessibility, health equity, hospice and palliative care, NIMBY syndrome, South Korea, structural determinants of health

## Abstract

**Introduction:**

East Asia is experiencing an unprecedented demographic transformation, with rapidly aging populations creating an urgent public health demand for accessible hospice and palliative care facilities. However, infrastructural planning regulations may implicitly marginalize these essential health services as stigmatized facilities, creating regulatory and institutional conditions that can constrain community-adjacent access to end-of-life care.

**Methods:**

This study conducts a structured comparative legal and health policy analysis of facility-siting frameworks in South Korea and China. It combines national-level legal and policy analysis with illustrative city-level cases of Seoul and Beijing to examine how divergent structural systems—market-based land acquisition in South Korea and state-led public welfare allocation in China—shape the regulatory conditions for geographic accessibility in end-of-life care. The analysis draws on statutory texts, public health policy documents, descriptive infrastructure indicators, and documented social resistance, including NIMBYism.

**Results:**

South Korea’s hospital-centered facility classification and market-based siting system may create regulatory conditions that constrain community-adjacent hospice access. Hospice-compatible facilities are generally embedded within hospital-oriented land-use categories, while high land costs, zoning restrictions, greenbelt constraints, and neighborhood opposition may further limit residential-adjacent development. By contrast, China’s administrative land allocation, medical-nursing integration policies, and community health planning create a more explicit formal pathway for embedding hospice-compatible services within community health networks. Nevertheless, this pathway remains constrained by fragmented reimbursement, uneven workforce capacity, variable facility readiness, regional implementation disparities, and persistent rural–urban inequalities.

**Conclusion:**

Facility-siting rules, health financing systems, workforce distribution, regional disparities, and culturally rooted resistance to death-adjacent facilities jointly shape the accessibility of hospice and palliative care in South Korea and China. Achieving health equity in end-of-life care therefore requires moving beyond clinical guidelines to address the structural and geographical determinants of facility distribution. Legal and planning reforms should be accompanied by financing support, workforce development, stakeholder engagement, and local implementation mechanisms to integrate end-of-life care infrastructure more effectively into community health planning.

## 1 Introduction

East Asia stands at the precipice of an unprecedented demographic transformation (1, 2). The region’s “Silver Tsunami”—a metaphor capturing the overwhelming force of population aging—is reshaping healthcare systems, social structures, and urban landscapes in ways that policymakers are only beginning to comprehend. South Korea crossed the threshold into a “super-aged society” in December 2024, with its older adults population exceeding 20% of the total population, while China’s population aged 60 and above is projected to exceed 400 million around 2035 (3). This demographic shift carries a profound yet underexamined spatial dimension: as death rates inevitably rise with aging populations, the question of where people die—and where they can access dignified end-of-life care—emerges as a critical urban planning challenge.

The concept of “spaces of dying,” introduced by geographers studying deathscapes, draws attention to the physical locations where end-of-life care occurs and the socio-spatial processes that determine their distribution. In contemporary East Asia, these spaces are increasingly contested terrain. Hospice and palliative care facilities, despite their essential role in providing comfort to terminally ill patients and their families, are frequently classified as locally unwanted land uses (LULUs) and subjected to intense NIMBY (Not In My Backyard) opposition. Residents associate such facilities with death, misfortune, and declining property values, triggering conflicts that can derail construction projects or relegate facilities to peripheral locations far from the communities they serve.

Both South Korea and China have enacted substantive legislation governing hospice and palliative care. South Korea’s Act on Hospice and Palliative Care and Decisions on Life-Sustaining Treatment (2016) established a comprehensive framework for end-of-life care services, while China’s Basic Medical and Health Promotion Law (2019) mandated hospice care as a component of medical institutions’ service portfolios. However, these laws focus primarily on clinical standards, patient eligibility, and consent procedures—leaving the spatial dimension of hospice care largely unaddressed. The critical question of where hospice facilities can be built, and how land use planning regulations affect their accessibility, remains subordinate to broader medical facility zoning frameworks that were not designed with end-of-life care in mind.

This regulatory gap can be understood through Edward Soja’s concept of “spatial injustice” (4), which draws attention to how geographic arrangements and institutional rules may contribute to unequal access to essential services (5, 6). When zoning laws restrict hospice facilities to industrial zones, greenbelt peripheries, or existing hospital complexes, they may reduce the feasibility of receiving end-of-life care in familiar community settings. The principle of “aging in place,” endorsed by the World Health Organization’s Age-Friendly Cities framework (7), extends logically to “dying in place”—yet land use planning rarely accommodates this extension (8).

This study addresses this gap through a structured comparative legal and health policy analysis of hospice facility siting frameworks in South Korea and China. The comparison focuses on three questions: how hospice-compatible facilities are legally classified, how land-use and planning rules shape their possible locations, and how community-level implementation conditions affect spatial accessibility. South Korea and China offer instructive contrasts because they combine similar demographic pressures with different land tenure, planning, and facility-siting systems. Rather than treating national policy frameworks as self-executing, this study examines how these frameworks operate through specific regulatory mechanisms and city-level implementation contexts. By combining national legal-policy analysis with illustrative cases from Seoul and Beijing, the study identifies the regulatory conditions under which end-of-life care infrastructure becomes more or less feasible within residential communities.

## 2 Methods and materials

This study employs a comparative legal analysis methodology to examine land use planning frameworks governing hospice facility siting in South Korea and China. Comparative legal analysis is particularly suited to this inquiry because it enables systematic identification of functional equivalents and structural differences across legal systems (9) while illuminating how divergent regulatory approaches produce distinct spatial outcomes (10). No human subjects research was conducted; all data derive from publicly available legal texts, government policy documents, and secondary scholarly sources, thereby obviating the need for institutional ethics approval. This study was designed as a structured comparative legal and health policy analysis rather than as a clinical, epidemiological, or GIS-based accessibility study. The comparison was guided by three research questions. First, how do South Korea and China legally classify and regulate hospice and palliative care facilities for land-use and facility-siting purposes? Second, what institutional mechanisms facilitate or constrain the location of hospice-compatible services near residential communities? Third, how do differences in land acquisition, zoning permission, community-embedded service pathways, social resistance, and implementation capacity shape the regulatory conditions for spatial accessibility?

Primary legal sources were retrieved from official government databases. For South Korea, the Korea Ministry of Government Legislation (MOLEG) database provided access to the National Land Planning and Utilization Act, the Building Act, and the Act on Hospice and Palliative Care and Decisions on Life-Sustaining Treatment, including all amendments through 2024. For China, the National People’s Congress (NPC) database and Peking University Law Information Platform (Beida Fabao) supplied the Land Administration Law, Urban–Rural Planning Law, and relevant State Council policy documents. Supplementary policy materials were obtained from the Korean Ministry of Health and Welfare (MOHW) and China’s National Health Commission (NHC).

The search strategy employed both English and native-language keywords. Korean-language searches used terms including “호스피스” (hospice), “완화의료” (palliative care), “용도지역” (zoning), “의료시설” (medical facility), and “개발제한구역” (development restriction zone/greenbelt). Chinese-language searches employed “安宁疗护” (hospice care), “医养结合” (medical-nursing combined), “划拨用地” (allocated land), “控制性详细规划” (regulatory detailed planning), and “15分钟生活圈” (15-min life circle). Statistical data on population aging and hospice infrastructure were obtained from Statistics Korea (KOSIS), the Korean National Hospice Center, and China’s National Bureau of Statistics.

Documents were included if they met at least one of the following criteria: (1) they directly regulated hospice care, palliative care, medical facilities, nursing hospitals, older-adult care institutions, community health facilities, or land-use planning; (2) they specified facility classification, zoning permission, land allocation, adaptive reuse, public-service infrastructure requirements, or community-level health service standards; (3) they provided official statistical or policy information on population aging, hospice infrastructure, medical-nursing integration, or pilot hospice programs; or (4) they documented social resistance, NIMBY conflicts, or implementation barriers relevant to older-adult care, hospice-compatible services, or death-adjacent facilities. Documents were excluded if they addressed only clinical treatment standards without relevance to facility siting, focused only on individual consent or life-sustaining treatment decisions without spatial or institutional implications, duplicated policy summaries without independent regulatory content, or consisted of media reports that could not be connected to official documents, court cases, municipal materials, or academic sources.

The analytical framework combined doctrinal legal analysis, policy text content analysis, and structured cross-jurisdictional comparison (11). The comparison proceeded in four steps. First, doctrinal legal analysis was used to identify the formal legal categories governing hospice-compatible facilities, including whether such services are classified as hospitals, nursing hospitals, welfare facilities, community health functions, or mixed medical-nursing institutions. Second, policy text content analysis was used to examine how national and local policy documents address hospice infrastructure, land allocation, zoning permission, adaptive reuse, community health planning, and implementation support (12). Third, the two jurisdictions were compared across six common analytical dimensions: facility classification, siting permission, land acquisition mechanism, community-embedded delivery pathway, documented social resistance, and implementation capacity. These dimensions correspond to the operational indicators summarized in Table 1. Fourth, documented NIMBY cases and the Seoul–Beijing city-level cases were used for contextual triangulation, linking national legal frameworks to concrete implementation settings.

**Table 1:** Operational indicators for assessing geographic accessibility.

Indicator	Operational meaning in this study	Evidence used
Facility classification	Whether hospice care is classified as a hospital-based medical facility, welfare facility, or community health service function.	Statutory and regulatory classification of medical, welfare, and community health facilities.
Siting permission	Whether hospice-compatible facilities can be located in or near residential communities.	Zoning rules, planning standards, and permitted land-use categories.
Land acquisition mechanism	Whether land access depends mainly on market purchase, public allocation, adaptive reuse, or public-service planning.	Land-use law, allocation rules, and medical-nursing integration policy documents.
Community-embedded delivery pathway	Whether hospice care can be incorporated into existing community health or older-adult care institutions.	Community health center standards, medical-nursing integration policies, and city-level cases.
Social resistance	Whether death-adjacent or older-adult care facilities encounter documented neighborhood opposition.	Reported NIMBY cases, municipal surveys, and secondary literature.
Implementation capacity and contextual modifiers	Whether health financing, workforce distribution, facility readiness, quality governance, and rural–urban or regional disparities support or constrain actual service provision.	Hospice reimbursement policies, workforce and training policies, regional implementation documents, community health infrastructure standards, and pilot-program materials.

These indicators are used as structured proxy measures. They do not replace GIS-based accessibility analysis, but they reduce reliance on purely narrative interpretation by specifying how spatial accessibility is assessed across the two jurisdictions. For each jurisdiction, the analysis applied these indicators in the same sequence. The country-level sections first examine legal classification and siting permission, then land acquisition and planning mechanisms, followed by community-embedded delivery pathways, social resistance, and implementation capacity. Evidence was triangulated across legal texts, policy documents, descriptive infrastructure indicators, case materials, and secondary literature where available. This protocol does not eliminate the limitations of a document-based comparative study, but it provides a consistent procedure for identifying and comparing the regulatory conditions that shape hospice facility accessibility in both countries. Health financing systems, cultural attitudes toward end-of-life care, rural–urban disparities, and healthcare workforce distribution were treated as contextual modifiers rather than as variables controlled through statistical modeling. Because this study is a document-based comparative legal and health policy analysis, it does not estimate the independent causal effect of each factor. Instead, these factors are incorporated into the analysis to examine how they may strengthen, weaken, or condition the relationship between land-use regulation and hospice accessibility. This approach helps avoid interpreting spatial regulation as a single-cause explanation for hospice access. Triangulation in this study is used to strengthen interpretive consistency rather than to establish causal effects. Legal texts identify formal permissions and restrictions; policy documents show government priorities and implementation instruments; descriptive indicators provide background evidence on service capacity; NIMBY cases illustrate how siting conflicts may arise in practice; and the Seoul–Beijing cases contextualize the national-level comparison. The convergence of these sources supports mechanism-level interpretation, but it does not prove that zoning rules or NIMBY conflicts independently cause hospice inaccessibility.

The same comparative questions were applied to both South Korea and China: how is hospice-compatible care legally classified; where may such facilities or services be located; how is land obtained or allocated; whether community-embedded delivery pathways exist; whether social resistance affects siting; and whether financing, workforce, facility readiness, and quality governance support implementation. This procedure was intended to make the comparison transparent and reproducible, while recognizing that the study assesses regulatory and institutional accessibility conditions rather than directly measuring patient-level travel time or facility catchment areas.

To further connect the national-level legal and policy analysis with concrete implementation contexts, this study also incorporates two illustrative city-level cases: Seoul and Beijing. These cities were selected because both are major metropolitan areas facing population aging, land-use pressure, and rising demand for community-level health and older-adult care services, while operating under markedly different land-use and planning systems. Seoul represents a dense Korean metropolitan context in which market-based land acquisition, medical-facility classification, greenbelt constraints, and neighborhood resistance interact. Beijing represents a Chinese metropolitan context in which community health service centers, medical-nursing integration, and the 15-min life circle provide a formal pathway for embedding end-of-life care within neighborhood infrastructure. These cases are not intended as statistically representative samples, but as illustrative cases used to examine whether the mechanisms identified in the national-level analysis are visible in concrete city-level siting and accessibility dynamics.

Operationalization of geographic accessibility. Because comparable geocoded facility-level datasets and patient-level travel-time data were not available for both countries, this study does not conduct a full GIS-based accessibility analysis. Instead, geographic accessibility is operationalized through legal-policy proxy indicators that capture the regulatory and institutional conditions under which hospice and palliative care services can be located near residential communities.

These proxy indicators include: (1) facility classification, referring to whether hospice care is treated as a hospital-based medical facility, a welfare facility, or a community health service function; (2) siting permission, referring to whether hospice-compatible facilities can be located in or near residential areas; (3) land acquisition mechanism, referring to whether land is obtained through market purchase, public allocation, adaptive reuse, or public-service planning; (4) community-embedded delivery pathway, referring to whether hospice care can be incorporated into community health centers, nursing institutions, or medical-nursing integrated facilities; (5) documented social resistance, referring to NIMBY conflicts, stigma, or opposition to death-adjacent facilities; and (6) implementation capacity, referring to financing, workforce, facility readiness, and quality governance.

These indicators do not directly measure patient travel distance or travel time. Rather, they provide a structured way to assess whether each legal and policy system creates favorable or unfavorable conditions for spatially proximate hospice access. The analysis therefore treats geographic accessibility as a regulatory and institutional accessibility condition, rather than as a fully measured GIS outcome.

## 3 Legal framework in South Korea: addressing zoning restrictions and NIMBYism

South Korea’s regulatory framework for hospice facility siting operates through the interaction of three distinct legal domains: general land use zoning under the National Land Planning and Utilization Act, building classification under the Building Act, and substantive hospice service requirements under the Act on Hospice and Palliative Care. This tripartite structure creates a complex regulatory maze that developers must navigate, with each layer imposing independent constraints on facility location. The fundamental tension lies in the fact that these legal frameworks were developed at different times for different purposes, none of which specifically contemplated the unique spatial needs of hospice care.

**Figure 1:**
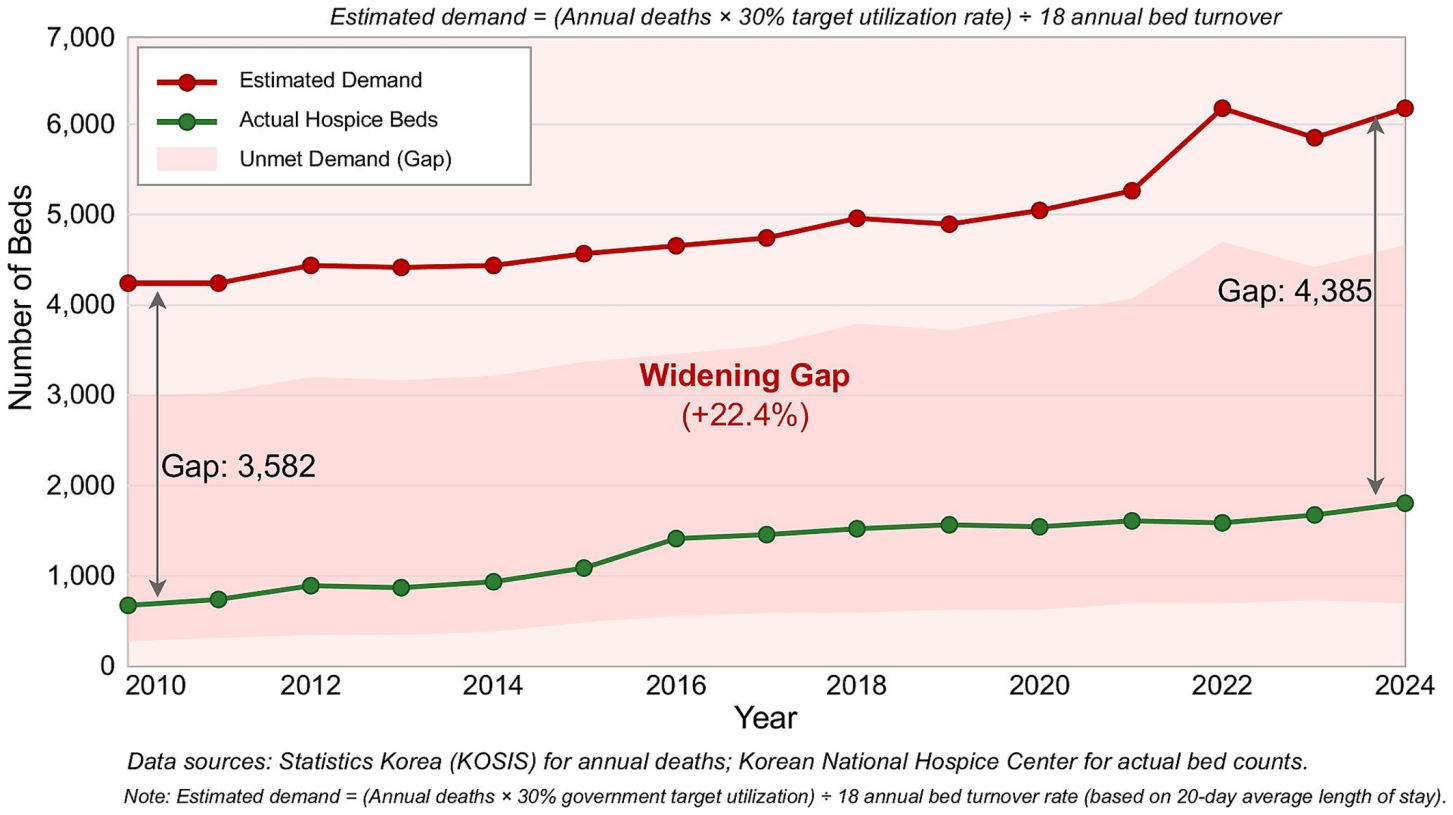
Estimated demand vs. actual supply of hospice beds in South Korea (2010–2024). The upper line represents estimated bed demand calculated as (annual deaths × 30% government target utilization rate) ÷ 18 annual bed turnover rate. The lower line shows actual hospice beds in designated institutions. The shaded area represents unmet demand. Despite substantial growth in actual beds (+169%), the gap widened from 3,582 beds in 2010 to 4,385 beds in 2024 (+22.4%), illustrating the supply–demand “scissors gap.” Data sources: Statistics Korea (KOSIS); Korean National Hospice Center.

Korea’s rapid transition to a super-aged society has dramatically intensified this tension. The proportion of the population aged 65 and above increased from 10.0% in 2008 to 19.2% in 2024, with projections indicating 40% older adults population by 2050. This demographic shift has far outpaced the expansion of hospice infrastructure. While the absolute number of hospice beds has grown significantly—from 282 beds across 19 institutions in 2008 to approximately 1,815 beds across 103 inpatient facilities by 2024—the per-capita availability remains grossly inadequate. The hospice utilization rate among eligible cancer patients has risen from 11.9% in 2014 to approximately 23% in 2024, still far below the government’s target of 30%. This supply–demand gap, illustrated in Figure 1, does not directly measure geographic accessibility or patient travel distance. However, it provides supply-side descriptive evidence that the expansion of hospice policy has not eliminated capacity constraints.

The figure is therefore not used as standalone evidence of spatial inequity, but as a descriptive starting point for examining whether legal and planning mechanisms may further affect accessibility. Building on this supply-side evidence, the following sections examine the legal and planning mechanisms that may further shape hospice facility distribution in South Korea. The analysis focuses on how Korean law classifies hospice facilities, where such facilities may be located, the constraints posed by greenbelt restrictions, and the role of NIMBY conflicts in facility siting.

### 3.1 Land use zoning for medical facilities

The National Land Planning and Utilization Act, enacted January 1, 2003 through the consolidation of prior urban and regional planning statutes, establishes the foundational zoning framework governing all land development in South Korea. Article 36 divides the national territory into four major categories: Urban Areas, Management Areas, Agricultural and Forestry Areas, and Natural Environment Conservation Areas. Within Urban Areas, the Act further distinguishes Residential Zones, Commercial Zones, Industrial Zones, and Green Zones, each subject to distinct development regulations specified in the Enforcement Decree’s Appendices 4 through 21.

Medical facilities, including hospitals capable of housing hospice units, face differential treatment across these zone types. The Semi-Residential Zone, designed to accommodate mixed residential and commercial uses, permits hospitals under Enforcement Decree Appendix 7 with maximum building coverage of 60% and floor area ratio of 500%. This zone represents the most permissive residential-adjacent classification for medical facility development. However, the same appendix has prohibited isolation hospitals in Semi-Residential zones since the 1980s—a restriction reflecting historical concerns about disease-related facilities that casts a regulatory shadow over death-related facilities including hospices.

The Building Act Enforcement Decree Appendix 1, most recently amended May 4, 2021, provides the classification system determining which facilities qualify as “medical facilities” subject to these zoning restrictions. Category 9 encompasses medical facilities, distinguishing between hospitals, general hospitals with 100 or more beds, and nursing hospitals with 30 or more nursing care beds. Crucially, hospice care units within hospitals are classified as part of the hospital category, meaning they inherit the hospital’s zoning permissions rather than constituting a distinct facility type. Standalone hospice facilities similarly fall under Category 9, requiring the same zoning permissions as hospitals—Semi-Residential, Commercial, or certain Neighborhood Commercial zones—with only conditional permission in Natural Green Areas and outright prohibition in Development Restriction Zones.

This classification structure creates a regulatory condition that may limit residential-adjacent hospice development: independent hospice facilities are less likely to fit within ordinary residential land-use categories, even though proximity to patients and families is especially important for end-of-life care. Unlike clinics, classified under Category 3 as “First-Class Neighborhood Living Facilities” and permitted in most residential zones for outpatient services, hospice facilities providing inpatient care are channeled toward commercial centers or urban peripheries. The regulatory logic treating hospice care as equivalent to general hospital services ignores the distinctive characteristics of end-of-life care—the need for family proximity, frequent visitation, and integration with community support networks—that argue for residential-adjacent siting.

Table 2 summarizes the relationship between facility types, their legal classifications, and permitted zoning categories under Korean law. The table reveals how the Building Act’s category system, combined with the National Land Planning and Utilization Act’s zoning framework, constrains the spatial options for hospice facility development.

**Table 2:** Definitions and permitted zones for hospice facilities in Korean law.

**Facility type**	**Building act classification**	**Key zoning permissions**	**Minimum requirements**
General hospital	Category 9 medical facility	Semi-residential, commercial; conditional in general residential 3	100 + beds with specified departments
Hospital/Nursing hospital	Category 9 Medical Facility	Semi-residential, commercial, neighborhood commercial; limited in natural green	30 + beds (hospital) or 30 + nursing care beds
Clinic	Category 3 first-class neighborhood living facility	Permitted in most residential and commercial zones	Outpatient services only
Standalone hospice	Category 9 medical facility (hospital classification)	Same as hospital; prohibited in development restriction zones	Must meet Article 25 designation criteria per Ministerial Decree

Hospice care units within existing hospitals are classified under the host hospital’s category. Natural Green Area limits: 20% building coverage, 100% floor area ratio.

**Figure 2:**
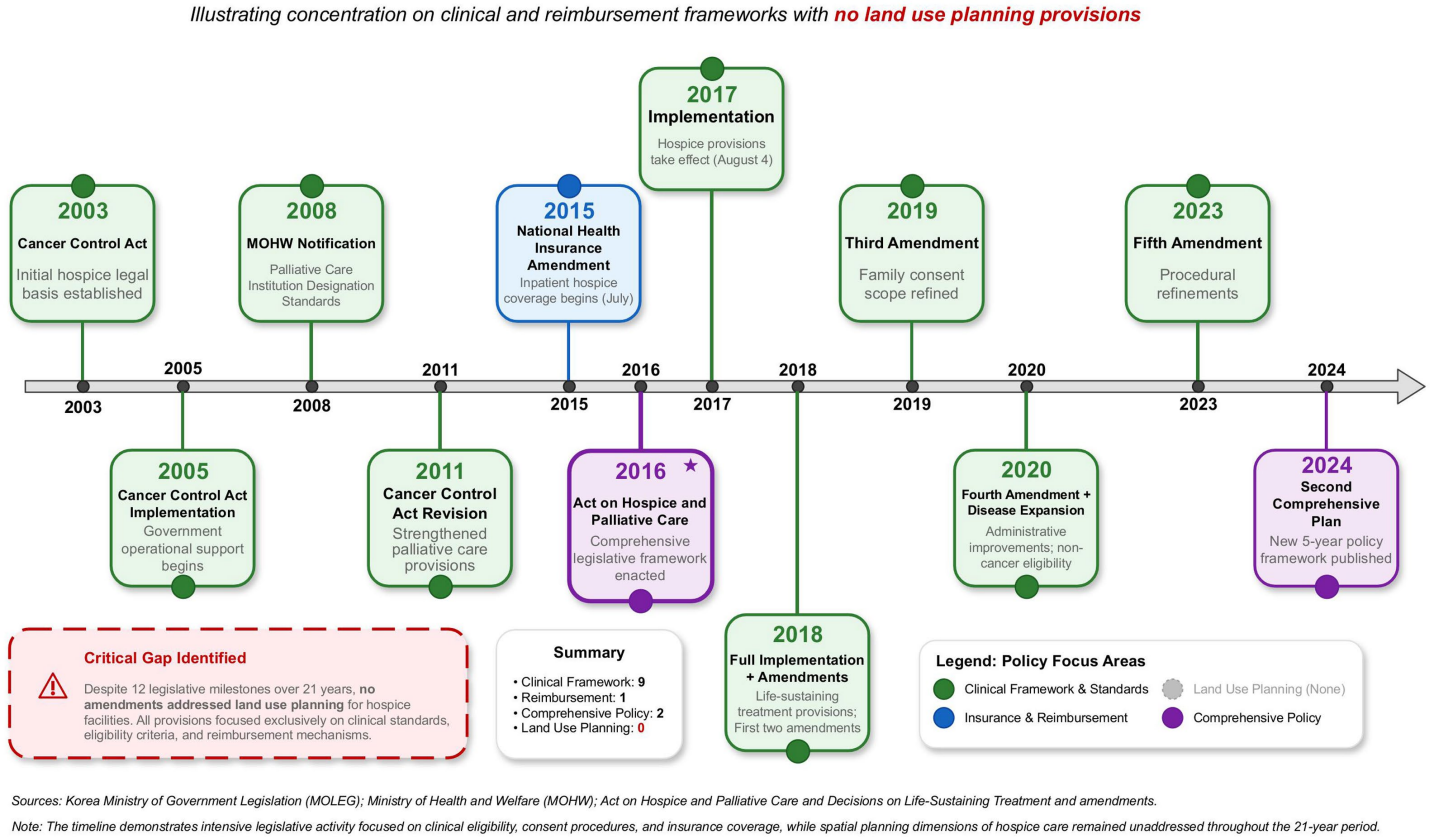
Timeline of legislative amendments and policy milestones regarding hospice and palliative care in South Korea (2003–2024). The timeline maps 12 major milestones across 21 years. Green indicates clinical or service-standard reforms, blue indicates reimbursement-related reforms, and purple indicates comprehensive hospice policy planning. No milestone directly amended land-use or zoning rules for hospice facility siting. Sources: Korea Ministry of Government Legislation (MOLEG); Ministry of Health and Welfare.

Figure 2 summarizes the major legislative and policy milestones in South Korea’s hospice and palliative care framework from 2003 to 2024. Across this period, reforms progressively expanded service eligibility, consent procedures, reimbursement arrangements, and national hospice planning. However, hospice-specific facility siting and land-use issues did not develop as a parallel reform track.

Figure 2 is therefore used as a descriptive policy-timeline indicator rather than as a direct accessibility measure. Its analytical value lies in showing that substantive hospice policy and spatial planning reform have followed separate trajectories. This sequencing gap helps explain why general land-use rules, rather than hospice-specific legislation, continue to shape where facilities may be located. The following section therefore examines greenbelt restrictions and siting conflicts as mechanisms through which this policy gap becomes relevant to facility accessibility.

### 3.2 Spatial conflicts and greenbelt restrictions

South Korea’s Development Restriction Zones, commonly known as greenbelts, represent the most restrictive category in the national land use hierarchy (13). Established under the Special Act on the Management of Development Restriction Zones, these zones encircle major metropolitan areas with bands of protected open space, covering approximately 3,800 to 3,980 square kilometers—roughly 4% of national territory (14). The greenbelt system, originally implemented in 1971 to prevent urban sprawl and preserve agricultural land, has evolved into a significant constraint on welfare facility development, including hospice care infrastructure (15).

Within Development Restriction Zones, new hospital construction is generally prohibited, with no specific exception carved out for hospice facilities. Public welfare facilities may receive limited exceptions under strict conditions, but the approval process is arduous and outcomes uncertain. The Ministry of Health and Welfare has acknowledged that some local governments returned allocated construction funds for older adults care facilities due to resident opposition in greenbelt-adjacent areas, prompting consideration of greenbelt land release specifically to site facilities away from residential opposition (16). This approach, however, may shift siting pressure toward peripheral areas rather than resolving the underlying problem of community-adjacent access; facilities built on released greenbelt land may still remain distant from many of the communities they are intended to serve (17).

These zoning constraints may have important implications for hospice accessibility, even though the present study does not directly measure facility distribution or patient travel distance. For nursing hospitals and long-term care facilities facing similar siting pressures, lower land costs and fewer regulatory barriers in peripheral or greenbelt-adjacent areas may create incentives for non-central locations. Such siting patterns, if not balanced by community-based service pathways, can make family visitation and continuity of care more difficult. The key analytical point is therefore not that zoning rules alone determine accessibility, but that they create institutional conditions that may make community-adjacent hospice provision more difficult.

NIMBY conflicts can compound these regulatory barriers by adding political and administrative uncertainty to otherwise legally permissible projects. The Inwae Dongsan case in Gwangju (2007–2008) provides instructive precedent (18). When developers sought to construct a 65-bed nursing facility in Bongseondong, adjacent apartment residents organized sustained opposition, ultimately triggering criminal prosecution. Two resident representatives were sentenced to 8 months imprisonment, suspended for 2 years, for obstruction of business, interference with public service, and traffic obstruction. Residents had specifically objected to a 3.7 pyeong funeral facility within the complex, citing traffic concerns and property value impacts—arguments that would apply with even greater force to standalone hospice facilities (19).

The Daegu Buk-gu case (2008) demonstrates that NIMBY opposition can succeed administratively where criminal liability fails to deter protesters. Facing intense resident opposition, the district office revoked the construction permit for a 60-bed nursing home—a rare instance of administrative capitulation to community pressure (20). Conversely, the Geumcheon Silber Center case in Seoul (2008–2010) illustrates prolonged conflict without resolution: adjacent apartment residents erected protest banners labeling the older adults care facility as a “NIMBY facility,” and district persuasion efforts extended over years (21).

Death-related facilities face particularly intense opposition. Crematorium and memorial park conflicts have generated some of Korea’s most contentious land use disputes. Gapyeong County experienced repeated failures in establishing regional crematoriums in 2006 and again in 2020–2022. The Hanam crematorium dispute triggered South Korea’s first mayoral recall vote—though ultimately unsuccessful, the episode demonstrated the political stakes involved in death-related facility siting. Seoul City’s 2023 survey on non-preferred facilities found funeral homes and columbarium facilities rated low on both necessity and preference dimensions. Top conflict factors included environmental, safety, and health concerns (21.1%), emotional harm from facility stigma (18.0%), and government’s unilateral approach (17.1%) (16). Notably, 82.1% of respondents supported compensation for affected residents, suggesting potential policy mechanisms for conflict resolution.

**Figure 3:**
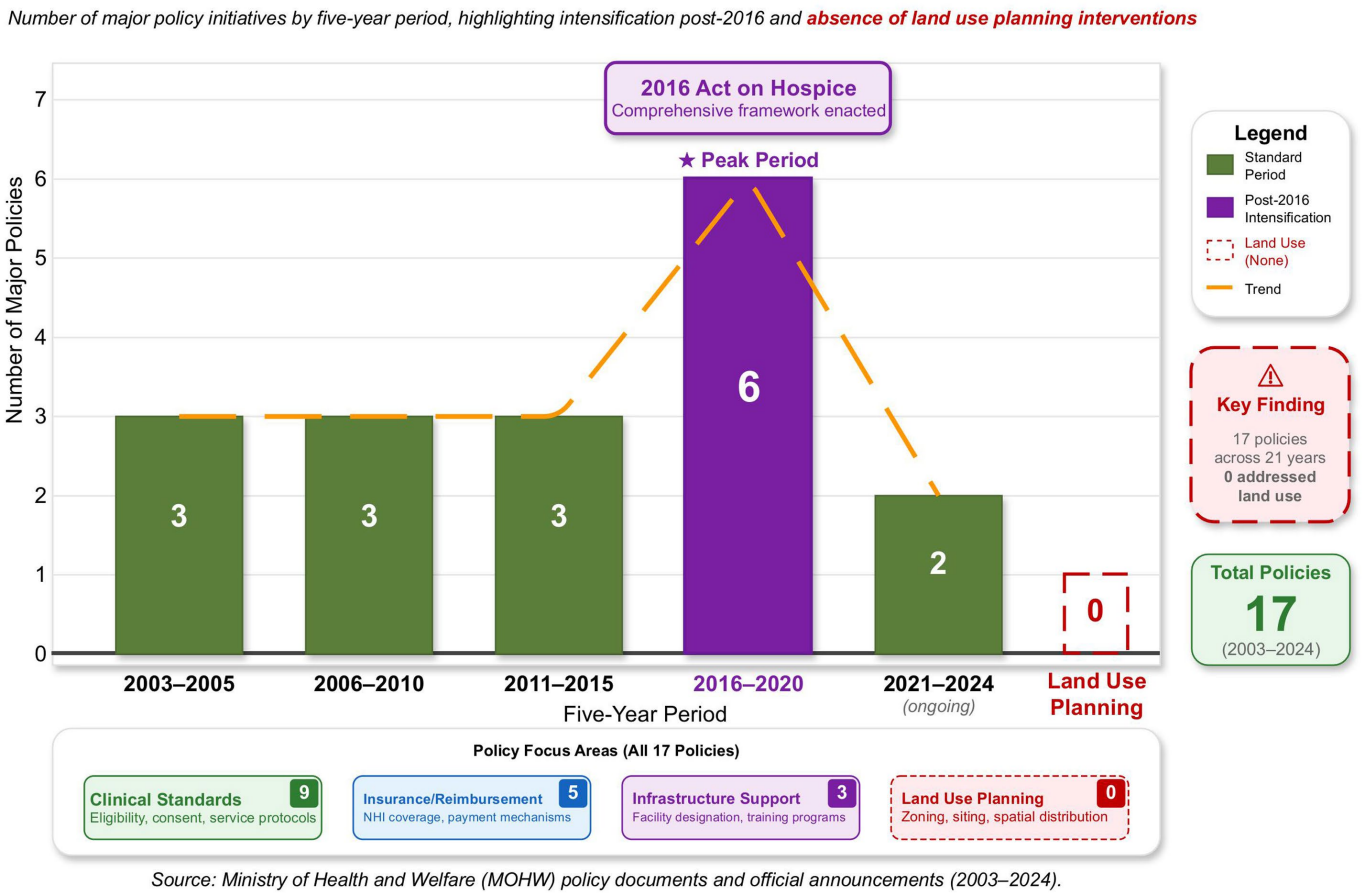
Government support policies for hospice infrastructure in South Korea (2003–2024). The bar chart displays the number of major policy initiatives by five-year period: 2003–2005 (3), 2006–2010 (3), 2011–2015 (3), 2016–2020 (6), and 2021–2024 (2). The intensification following the 2016 Act on Hospice and Palliative Care is evident, with policy output doubling in the 2016–2020 period. However, all 17 policies focused exclusively on clinical standards, insurance coverage, and infrastructure support—none addressed land use planning or zoning provisions for hospice facility siting. Source: Ministry of Health and Welfare (MOHW).

Figure 3 functions as a descriptive policy indicator rather than a direct accessibility measure. It shows that government support for hospice infrastructure intensified after the 2016 Act, particularly through National Health Insurance coverage expansion, home hospice pilot programs, and pediatric palliative care initiatives. However, the policy instruments identified in the figure remained concentrated on clinical standards, reimbursement, and institutional support, rather than land-use classification, zoning permission, or facility-siting reform. The analytical implication is that substantive hospice policy and spatial accessibility policy have developed unevenly, which helps explain why facility siting remains a separate regulatory problem.

Health financing and workforce capacity therefore modify, but do not replace, the land-use analysis in the Korean context. National Health Insurance coverage and designated hospice institutions may improve affordability, clinical standardization, and institutional viability, but payment coverage cannot ensure geographically proximate access when eligible facilities remain subject to hospital-centered classification and zoning constraints. Similarly, specialized hospice workforce capacity may support service quality within designated institutions, but it may also reinforce reliance on institution-based care rather than small-scale community-embedded models. Rural–urban differences further complicate this picture: metropolitan areas face high land costs and stronger siting conflicts, while non-metropolitan areas may face thinner service networks and longer travel burdens. These factors are therefore treated as contextual modifiers shaping how zoning and facility classification affect actual accessibility.

## 4 Approaches in China: centralized planning and integrated care

China’s approach to hospice facility siting differs from the Korean model because it operates within a socialist land tenure system and a more centralized planning tradition. In formal terms, Chinese institutions may access land through administrative allocation for qualifying public welfare purposes, which can reduce the land-cost barriers faced by non-profit medical and welfare facilities. However, this formal advantage should not be equated with actual accessibility. Administrative allocation depends on local government priorities, approval procedures, land-use restrictions, and the fiscal incentives of local authorities. The Chinese model therefore creates a potential pathway for community-embedded hospice infrastructure, but it also introduces implementation risks that differ from Korea’s market-based siting constraints.

The contrast extends beyond land acquisition mechanisms to encompass distinct planning philosophies. China’s urban planning system emphasizes top-down coordination through hierarchical plans—from national strategies through provincial frameworks to municipal master plans and regulatory detailed plans—with explicit requirements for public service facility provision at each level. The “15-minute Life Circle” concept, codified in national urban planning standards, requires health and older-adult care facilities to be considered within neighborhood-level service planning. Beyond service proximity, this community-embedded planning logic may also influence the social acceptance of sensitive care facilities by integrating them into familiar neighborhood infrastructure rather than presenting them as standalone NIMBY facilities. It therefore represents a planning-based approach that differs from Korea’s zoning-centered model (22, 23).

China’s hospice care system has developed rapidly through progressive pilot expansion. The first batch of pilot regions, designated in October 2017, included five cities and districts. By 2023, the program had expanded to 152 pilot regions across the country, with over 4,000 medical institutions establishing hospice care departments. The 14th Five-Year Healthy Aging Plan targets comprehensive hospice coverage across all pilot regions by 2025 and training of at least 5,000 professionals during the plan period. This expansion, however, has occurred primarily through embedding hospice services within existing medical and older adults care institutions rather than constructing standalone facilities—a pattern driven partly by land use considerations. These figures indicate policy expansion and institutional uptake, but they do not by themselves establish effective service availability, consistent quality, or equitable access across regions.

**Figure 4:**
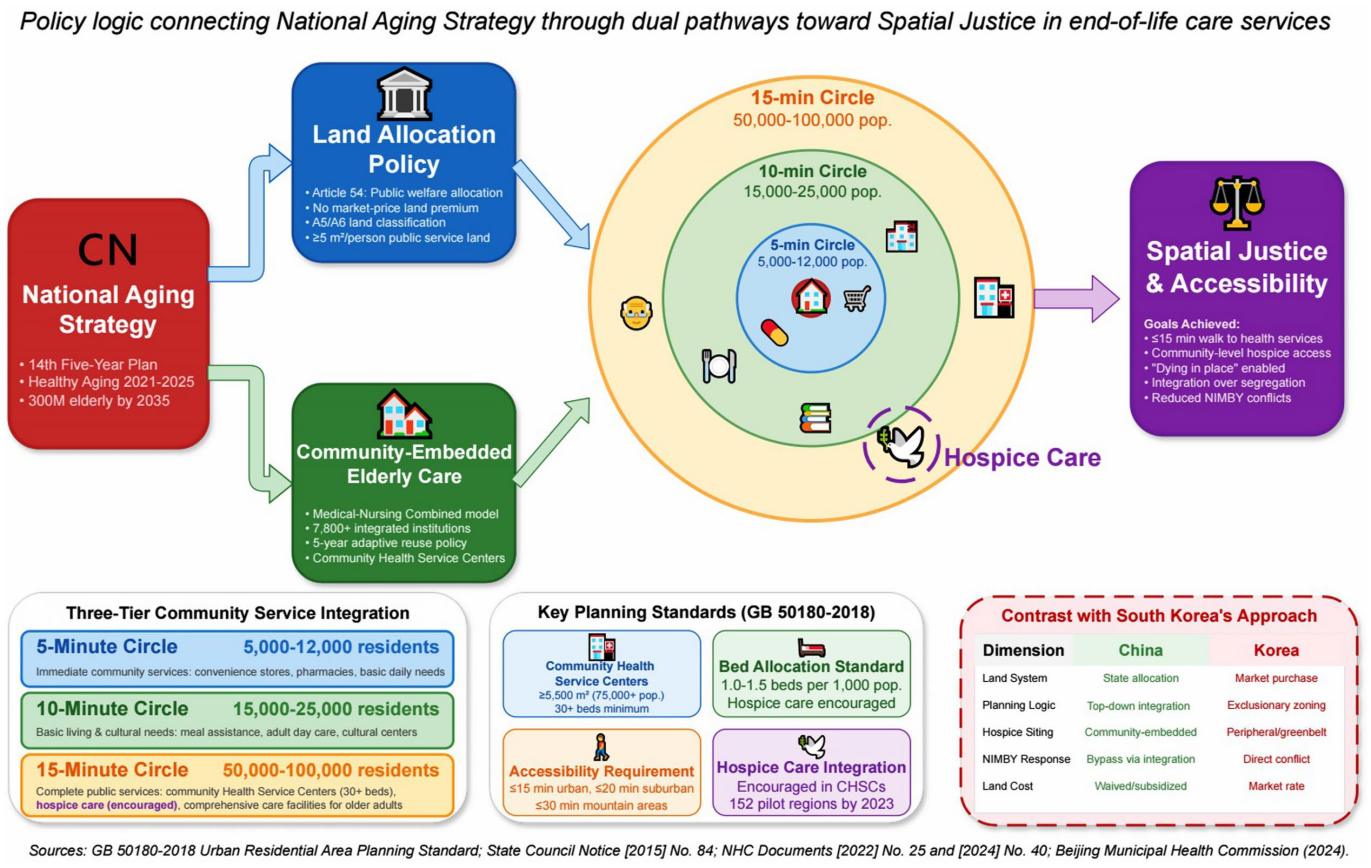
Conceptual framework of “15-minute Life Circle” incorporating older adults care in China. The diagram illustrates the policy logic connecting National Aging Strategy through two parallel pathways—Land Allocation Policy and Community-Embedded Older-adult Care—toward a formal planning objective of improving spatial accessibility in end-of-life care services. The concentric circles represent the three-tier community service integration: 5-min circle (5,000–12,000 population) for immediate services, 10-min circle (15,000–25,000 population) for basic living needs, and 15-min circle (50,000–100,000 population) for complete public services including Community Health Service Centers with hospice care. This framework represents a planning-based approach that differs from South Korea’s zoning-centered model, but it does not demonstrate that spatial accessibility has been achieved in practice. Sources: GB 50180–2018; State Council Notice [2015] No. 84; NHC Documents.

Figure 4 presents a conceptual framework for interpreting China’s community-embedded planning approach. It should be read as a policy-logic diagram rather than as empirical evidence that spatial justice has been achieved. The figure illustrates how national aging strategy, land allocation policy, and community-embedded service models are designed to support neighborhood-level access to older-adult and hospice-compatible services. Whether this policy logic produces actual accessibility depends on local implementation, financing, workforce capacity, and facility readiness, which are examined in the following sections.

### 4.1 Land administration law and public interest allocation

The Land Administration Law of the People’s Republic of China, comprehensively revised in 2019, establishes the legal foundation for land use in China’s socialist market economy. Article 54 articulates the core principle governing public welfare facility land acquisition: while state-owned land should generally be obtained through “compensated methods” including competitive bidding, auction, and listing, land for “urban infrastructure and public welfare undertakings” may be obtained through administrative allocation with approval from county-level or above people’s governments. This provision creates a dual-track land acquisition system with profound implications for hospice facility development.

Administrative land allocation enables qualifying institutions to receive land use rights without paying the substantial land premium that would otherwise be required in China’s urban real estate markets. For non-profit medical and welfare facilities, this mechanism can eliminate what is often the single largest capital expenditure in facility development. The 2019 amendment strengthened this framework by clarifying the scope of public welfare undertakings eligible for allocation and streamlining approval procedures. More precisely, Article 54 provides a general legal basis for administrative allocation of state-owned land for urban infrastructure and public welfare undertakings. The eligibility of medical, health, and social welfare facilities is further specified through land-allocation catalogues and sectoral policy documents.

The contrast with South Korea’s land acquisition framework is stark. Korean private hospitals and nursing facilities must purchase land through market transactions, competing with commercial developers willing to pay premium prices for well-located urban parcels. This market-based approach inherently disadvantages healthcare facilities, particularly those serving populations with limited ability to pay—exactly the demographic most in need of hospice care. Land costs often determine not merely facility quality but whether facilities can be developed at all in accessible locations. The result, as documented in Section 3, is concentration of hospice and long-term care facilities in peripheral areas where land costs are lower but accessibility is compromised.

China’s allocation system may mitigate this market-based barrier by reducing or removing land-cost pressures for qualifying public welfare facility development. However, the theoretical advantage requires effective implementation, and several practical constraints limit its realization. First, allocation decisions involve substantial administrative discretion; local governments may prioritize other land uses generating tax revenue over welfare facilities producing none. Second, allocated land typically carries use restrictions that limit subsequent adaptation, potentially creating inflexibility as service models evolve. Third, the allocation process itself can be time-consuming, delaying facility development even when approval is eventually granted.

Recent policy documents have sought to strengthen land support for older adults care and medical-nursing combined facilities. The State Council’s 2022 document on promoting medical-nursing integration (NHC Document [2022] No. 25) specifically addresses land use policy, clarifying that both medical/health land and social welfare land categories can accommodate medical-nursing combined projects. The document further introduces a five-year transitional policy permitting conversion of existing commercial buildings, factories, schools, and training facilities for medical-nursing services without requiring immediate land use classification changes. This flexibility enables adaptive reuse of underutilized urban properties for older adults care purposes, including hospice services, without triggering the full regulatory burden of new facility development.

The GB 50137–2011 Land Use Classification Standard provides the technical framework for planning implementation. Category A5 designates Medical and Healthcare Land, with subcategory A51 for Hospital Land and A53 for Special Medical Land including facilities for psychiatric and infectious disease treatment. Category A6 covers Social Welfare Land, encompassing nursing homes and related facilities. National planning standards mandate that combined A1-A6 public service land reach at least 5 square meters per person in urban planning. These standards, while not specific to hospice facilities, establish the spatial parameters within which end-of-life care infrastructure must be accommodated.

Taken together, China’s land-use framework should therefore be understood as enabling but not self-executing. It creates legal categories and allocation mechanisms that may support hospice-compatible infrastructure, but it does not guarantee that local governments will prioritize such facilities, that allocated land will be located near residents, or that community-level institutions will have the capacity to provide specialized palliative care.

### 4.2 The “medical-nursing combined” policy

China’s “Medical-Nursing Combined” policy represents a distinctive approach to integrating healthcare with older adults care services, with significant implications for hospice facility spatial planning. The policy framework, established through State Council General Office Notice [2015] No. 84, promotes deep integration of medical/healthcare services with older adults care/nursing services, recognizing that China’s rapidly aging population requires service models transcending traditional sectoral boundaries (24). For hospice care, this integration may offer a pathway to expand service availability without requiring construction of standalone facilities, by relying on functional diversification of existing institutions (25).

The medical-nursing combined model operates through multiple implementation pathways. First, nursing homes may add medical service capabilities, including basic healthcare, chronic disease management, and end-of-life care. Second, hospitals may establish geriatric wards or long-term care units serving patients requiring extended nursing support. Third, new facilities may be designed from inception to provide integrated services, avoiding the compartmentalization that characterizes traditional service models. Fourth, and most relevant for hospice care spatial planning, community health service centers and stations may expand their service portfolios to include palliative and hospice care, embedding end-of-life services within existing community infrastructure (26).

Land use policy explicitly supports this integration model. The 2022 policy document clarifies that facilities providing medical-nursing combined services can be sited on land classified for either medical/health use or social welfare use, removing a potential regulatory barrier that might otherwise require reclassification. More significantly, the transitional policy permitting adaptive reuse of commercial buildings, factories, and other structures enables medical-nursing combined facilities to occupy urban locations that would otherwise be unavailable for new healthcare construction. This flexibility parallels urban densification strategies employed in European contexts, where repurposing existing buildings for healthcare use has enabled service expansion without triggering the land use conflicts associated with new development.

The spatial implications of the medical-nursing combined model differ fundamentally from approaches requiring purpose-built hospice facilities. Rather than relying only on standalone hospice institutions, the policy allows incremental service expansion within existing facilities already embedded in community infrastructure. This approach may reduce the visibility of hospice care as a separate death-associated facility, but it can also obscure whether existing institutions have sufficient space, staffing, clinical protocols, and family-support environments for end-of-life care. This integration strategy may reduce some siting conflicts by expanding services within existing institutions, but it does not eliminate the need to assess whether such institutions have sufficient capacity, staffing, and quality safeguards for hospice care.

Available policy and infrastructure data indicate substantial institutional uptake of the medical-nursing combined model. By 2024, China had established over 7,800 medical-nursing combined institutions, with approximately 2 million beds providing various levels of integrated care. The number of nursing home beds with medical service capabilities has expanded significantly under supportive policies (27). For hospice care specifically, the pilot program expansion to 152 regions has occurred primarily through integration within existing institutions—community health centers, nursing homes, and hospital geriatric departments—rather than through standalone hospice facility construction (28).

However, the integration model carries its own limitations. Service quality may suffer when hospice care is added to institutions whose primary mission lies elsewhere; staff may lack specialized palliative care training, and facility design may inadequately accommodate end-of-life care needs. The policy emphasis on integration may inadvertently discourage development of specialized hospice facilities that could provide higher-quality care for patients with complex needs. Furthermore, the model’s reliance on existing institutional capacity means that service availability remains contingent on the geographic distribution of nursing homes and community health centers—a distribution that itself reflects historical planning decisions and market dynamics not optimized for hospice care access.

Recent policy evolution has sought to address quality concerns while maintaining the integration framework. The 2024 policy document (NHC Document [2024] No. 40) focuses specifically on quality management in medical-nursing combined facilities, establishing standards for staff training, service protocols, and outcome monitoring. Provincial implementation guidelines have further specified physical requirements for hospice care units within integrated facilities, including bed configurations, family accommodation provisions, and spiritual care spaces. These refinements suggest policy learning from early implementation experience, though comprehensive evaluation of quality outcomes remains limited.

The financial sustainability of hospice care within the medical-nursing combined model represents an ongoing challenge. Unlike Korea, where National Health Insurance coverage for inpatient hospice care has been available since 2015, China’s hospice care reimbursement remains fragmented across regions and insurance schemes. The 2017 pilot program included exploration of payment mechanisms, but national standardization has not been achieved. Without reliable reimbursement, institutions face disincentives to add hospice services, potentially limiting the spatial expansion that the policy framework otherwise enables (29). Recent policy documents have called for strengthening basic medical insurance coverage for hospice care and exploring long-term care insurance mechanisms, but implementation varies substantially across regions.

### 4.3 Urban master planning and community access

China’s urban planning system operates through a hierarchy of plans descending from national spatial strategies through provincial and municipal master plans to regulatory detailed plans that govern specific development parcels. The Urban–Rural Planning Law, revised in 2019, establishes the legal framework for this system, mandating that urban master plans allocate land for public service facilities including healthcare and older adults care. Regulatory detailed plans, governed by Ministry of Housing and Urban–Rural Development Order No. 7 (2010), translate master plan allocations into binding development controls specifying permitted uses, density parameters, and infrastructure requirements for individual parcels.

The “15-minute Life Circle” concept, codified in GB 50180–2018 Urban Residential Area Planning Design Standard, represents China’s most significant recent innovation in community-level service planning. The standard defines this circle as residential areas where “residents can satisfy material and living cultural needs within fifteen minutes of walking,” serving populations of 50,000 to 100,000 people bounded by urban arterial roads (30). Within this framework, the standard mandates inclusion of health services and older-adult care or disability assistance among community service facilities, creating a planning basis for hospice-compatible functions at the neighborhood scale.

The 15-min Life Circle framework establishes three tiers of community services with distinct population thresholds and facility requirements. The 15-min circle itself (50,000–100,000 population) requires complete public services including community health services. The 10-min circle (15,000–25,000 population) addresses basic living and cultural needs. The 5-min circle (5,000–12,000 population) provides immediate community service facilities. This tiered structure is designed to distribute health and older-adult care services across urban areas rather than concentrate them in specialized districts—a spatial logic fundamentally different from Korea’s exclusionary zoning approach (31).

Community Health Service Centers serve as the primary delivery mechanism for community-level health services within the 15-min Life Circle framework. Beijing’s 2024 revised standards illustrate the evolving expectations for these institutions. Centers serving populations over 75,000 must have minimum floor area of 5,500 square meters, with bed allocation of 1.0 to 1.5 beds per 1,000 population and a minimum of 30 beds per center. Crucially, the standards encourage centers to include beds for rehabilitation, nursing, and hospice care, explicitly recognizing end-of-life care as a community health service function. Accessibility standards mandate that centers be reachable within 15 min walking in urban areas, 20 min in suburban plains, and 30 min in mountain areas.

The September 2025 policy document “Urban 15-Minute Convenient Life Circle Construction Three-Year Action Plan” advances this framework by targeting establishment of 10,000 convenient life circles by 2030 across 100 pilot cities. The plan designates older adults care and meal assistance networks as mandatory components, reinforcing the spatial integration of aging services within community infrastructure. For hospice care, this policy trajectory may support the gradual incorporation of end-of-life services into community functions, but it does not by itself determine whether such services will be adequately financed, staffed, or clinically integrated.

The community-embedded approach to hospice care offers potential advantages for addressing NIMBY dynamics. When hospice services operate within familiar community health institutions already accepted by residents, they may reduce the visibility of hospice care as a stigmatized standalone facility and thereby lessen some forms of neighborhood opposition. Previous research on NIMBY attitudes toward community aging care service centers in China has found that resident opposition correlates with perceived risk, superstitious belief, and limited knowledge about services (32). Embedding hospice care within multi-function community health centers may dilute these concerns by associating end-of-life services with familiar, non-threatening healthcare activities.

However, community-level planning faces implementation challenges that temper its theoretical advantages. Local governments may underinvest in community health infrastructure relative to prestigious hospital projects that generate greater political visibility. Community health centers in many areas lack the staffing, equipment, and facility design to provide quality hospice care even when policy frameworks encourage such services. The gap between planning standards and implementation reality is particularly pronounced in less-developed regions and older urban areas where retrofitting existing facilities is difficult. Furthermore, while the 15-min Life Circle concept has received substantial policy attention, comprehensive evaluation of its impact on health service accessibility—let alone hospice care specifically—remains limited.

Regional disparities in hospice care availability persist despite the community planning framework. Pilot program coverage, while expanded to 152 regions, remains concentrated in economically developed coastal provinces and major cities. Rural areas, where population aging is often most acute due to youth outmigration, typically lack the community health infrastructure that the 15-min Life Circle concept presupposes. The policy framework’s urban orientation may inadvertently exacerbate spatial inequities in hospice access, contradicting the spatial justice principles it ostensibly serves. Accordingly, rural–urban disparity is treated here as a contextual modifier of China’s community-embedded planning model: formal proximity standards may have different practical meanings depending on whether local communities possess adequate primary health infrastructure, trained personnel, and stable reimbursement channels.

The Chinese case therefore should not be read as a straightforward countermodel to South Korea. Its formal planning instruments create more visible pathways for community-level embedding, but the effectiveness of those pathways depends on local fiscal priorities, reimbursement arrangements, workforce distribution, facility readiness, and the uneven capacity of community health institutions. The main asymmetry between the two systems is therefore not that one produces barriers while the other produces access, but that they generate different bottlenecks: Korea’s bottleneck is concentrated in legal classification, land cost, zoning, and resident opposition, whereas China’s bottleneck lies more in implementation capacity, service quality, financing, and regional inequality.

## 5 City-level illustrative case studies: Seoul and Beijing

### 5.1 Case selection and analytical purpose

The preceding sections examined national-level legal and policy frameworks governing hospice and palliative care facility siting in South Korea and China. To move the analysis from a general national comparison to a more specific examination of policy implementation, this section introduces two illustrative metropolitan cases: Seoul and Beijing. The cases are used to contextualize and triangulate the national-level analysis by examining how land-use classification, siting constraints, community delivery pathways, and implementation capacity operate in concrete urban settings.

Seoul was selected because it represents a dense urban environment in which land scarcity, high land prices, greenbelt pressure, medical-facility zoning, and neighborhood resistance converge. It therefore provides a useful case for examining how Korea’s national zoning and facility-classification framework operates in a concrete metropolitan setting. Beijing was selected because it provides a prominent example of China’s community-embedded planning model, including community health service centers, medical-nursing integration, and the 15-min life circle. Together, the two cases allow the analysis to move beyond a general national comparison and examine how different spatial governance models create distinct accessibility pathways and implementation constraints.

The cases are illustrative rather than statistically representative. Their purpose is not to establish generalizable causal effects across all Korean and Chinese cities, but to examine whether the mechanisms identified in the national-level analysis are visible in concrete urban settings.

### 5.2 Seoul: zoning pressure, greenbelt constraints, and community resistance

Seoul illustrates how a formally developed hospice and palliative care policy framework may still face city-level siting barriers when land-use regulation, market-based land acquisition, and community opposition intersect. As the capital and largest metropolitan area of South Korea, Seoul is characterized by high population density, intense competition for urban land, and long-standing tension between welfare-facility needs and neighborhood resistance to sensitive facilities. These conditions make it a useful city-level case for examining whether national hospice policy can translate into community-level spatial accessibility.

Under Korea’s legal framework, hospice facilities are not treated as a distinct land-use category designed around end-of-life care. Hospice units within hospitals inherit the legal classification of the host hospital, while standalone hospice facilities generally fall within the broader medical-facility classification. This means that hospice facilities are subject to zoning rules designed primarily for hospitals and other medical institutions, rather than for small-scale, family-oriented, community-adjacent end-of-life care. In a dense city such as Seoul, this classification matters because residential proximity is precisely what hospice care requires, yet residentially embedded locations are also the most politically and economically contested.

The Geumcheon Silber Center dispute illustrates how community resistance can operate at the city level. In that case, adjacent apartment residents opposed the establishment of an older-adult care facility and displayed protest banners describing the facility as a “NIMBY facility.” Although the facility was not a standalone hospice institution, the dispute is directly relevant because older-adult care, nursing care, and death-adjacent welfare facilities often trigger similar neighborhood concerns about stigma, property values, traffic, and emotional discomfort. The prolonged persuasion efforts by the district government show that legal permission alone does not necessarily produce social acceptance or practical siting feasibility.

The Seoul case therefore supports a more cautious interpretation of the Korean evidence. It does not prove that zoning rules alone cause inequitable hospice access. Rather, it illustrates a plausible mechanism through which medical-facility classification, high urban land costs, greenbelt pressure, and community opposition may jointly constrain the spatial embedding of end-of-life care. In such a context, hospice accessibility depends not only on clinical authorization or insurance coverage, but also on whether land-use law creates a feasible pathway for small-scale, community-adjacent facilities and whether local governments can manage resident concerns through consultation, compensation, and public education.

### 5.3 Beijing: community-embedded planning and implementation constraints

Beijing provides a contrasting city-level case for examining how China’s community-embedded planning approach may create formal pathways for neighborhood-level hospice access. Unlike Seoul, where hospice facilities are largely filtered through hospital-based classification and market-based land acquisition, Beijing illustrates how community health infrastructure can be used as a platform for integrating end-of-life care into ordinary neighborhood services.

The key institutional mechanism is the community health service center. Within the 15-min life circle framework, community health service centers are expected to provide accessible primary health and older-adult care functions within walking distance of residential areas. Beijing’s revised standards for community health service centers illustrate this model in concrete terms. Centers serving populations over 75,000 are required to have a minimum floor area of 5,500 square meters, with bed allocation of 1.0 to 1.5 beds per 1,000 population and a minimum of 30 beds per center. The standards also encourage centers to include beds for rehabilitation, nursing, and hospice care, thereby recognizing end-of-life care as a potential community health service function rather than only a specialized hospital-based service.

This model has important spatial implications. By embedding hospice-compatible services within existing community health institutions, Beijing’s approach may reduce the need to construct separate death-associated facilities that are more likely to trigger neighborhood opposition. Community health service centers are already familiar to residents and are generally understood as ordinary public service infrastructure. When hospice care is incorporated into such institutions, the service may become less visible as a stigmatized standalone facility and more acceptable as part of a broader continuum of aging, chronic disease, rehabilitation, and nursing care.

However, the Beijing case also shows why formal planning integration should not be equated with actual accessibility or service quality. A planning standard that authorizes or encourages hospice beds does not guarantee that community health service centers have the trained personnel, reimbursement mechanisms, physical space, or clinical capacity required for high-quality palliative care. The availability of hospice-compatible beds may also vary across districts, especially between central urban areas, suburban plains, and mountainous areas. Moreover, the 15-min life circle primarily establishes a spatial planning standard; it does not by itself resolve workforce shortages, fragmented financing, or uneven implementation across local institutions.

The Beijing case therefore supports a moderated conclusion. China’s planning framework creates a more explicit formal pathway for community-embedded hospice access, but this pathway differs from Korea’s hospital-centered and zoning-constrained model rather than simply overcoming it. Yet this pathway remains conditional on implementation capacity, financing, workforce training, and quality governance. Beijing demonstrates that community planning can make hospice access more spatially feasible, but it does not automatically make such access equitable, specialized, or clinically adequate.

In the absence of comparable GIS-based datasets, Table 3 uses city-level proxy indicators to compare the regulatory and institutional conditions for spatial accessibility in Seoul and Beijing. Table 3 summarizes the main city-level mechanisms through which the Seoul and Beijing cases illustrate different pathways and constraints in hospice accessibility. A more detailed analytical matrix is provided in Supplementary Table S1.

**Table 3:** City-level comparison of hospice accessibility mechanisms in Seoul and Beijing.

**Dimension**	**Seoul / Seoul metropolitan area**	**Beijing**
Siting pathway	Hospital-centered classification; market-based land acquisition; limited flexibility for small-scale community-adjacent hospice facilities.	Community health service centers and medical-nursing integration provide a formal pathway for neighborhood-level hospice-compatible services.
Spatial barrier	High land cost, zoning constraints, greenbelt pressure, and neighborhood opposition may restrict community-adjacent siting.	Planning standards support proximity, but actual access depends on district-level implementation, facility capacity, and service readiness.
Social acceptance	Death-adjacent and older-adult care facilities may trigger NIMBY resistance, as illustrated by the Geumcheon dispute.	Embedding hospice care within familiar community health institutions may reduce visible siting conflict, but cultural stigma may persist.
Main implication	Formal hospice policy does not automatically produce spatial accessibility.	Community planning creates an access pathway, but does not automatically ensure quality or equitable implementation.

The comparison suggests that spatial accessibility is shaped not by hospice legislation alone, but by the interaction between land-use governance, institutional design, social acceptance, and implementation capacity.

### 5.4 Cross-case synthesis

The Seoul and Beijing cases clarify and qualify the national-level comparison by converting the country-level analysis into mechanism-based findings. Seoul shows that even where hospice and palliative care policy has developed through clinical standards, insurance coverage, and institutional designation, city-level accessibility may remain constrained if land-use law continues to classify hospice facilities through hospital-centered categories and if local opposition makes community-adjacent siting politically difficult. In this sense, the Korean case illustrates the limits of substantive health policy when it is not matched by spatial planning reform.

Beijing shows the opposite possibility and its limits. Community health service centers and the 15-min life circle create a formal pathway for embedding hospice-compatible services within neighborhood infrastructure. This pathway may reduce the visibility of hospice care as a stigmatized standalone facility and may make proximity more feasible. However, Beijing also demonstrates that spatial planning integration is not equivalent to actual service availability. Without adequate reimbursement, trained palliative care personnel, facility design, and quality control, community-embedded access may remain nominal or uneven.

This cross-case synthesis should be interpreted as a structured qualitative comparison rather than as a quantitative accessibility result. Taken together, the two city cases support three findings. First, legal authorization of hospice care does not automatically produce spatial accessibility. Second, community-embedded planning can improve the regulatory conditions for access, but only when supported by financing, workforce distribution, facility readiness, quality mechanisms, and rural–urban implementation capacity. Third, NIMBYism should not be treated as an isolated social attitude; it reflects culturally rooted perceptions of death-adjacent facilities and interacts with facility classification, land cost, institutional design, and local governance. These findings support a more cautious conclusion: spatial governance does not mechanically determine hospice accessibility, but it creates institutional conditions whose effects are modified by health financing, workforce capacity, cultural attitudes, and regional inequality.

## 6 Discussion

The national-level comparison and the Seoul–Beijing city cases together reveal two contrasting but incomplete regulatory approaches to hospice facility siting. South Korea’s system, characterized by private land ownership, market-based acquisition, and hospital-centered facility classification, provides relatively developed clinical and insurance frameworks for hospice care, but the Seoul case illustrates how zoning constraints, greenbelt pressure, land cost, and neighborhood resistance may continue to restrict community-adjacent siting. China’s system, characterized by state land ownership, administrative allocation, medical-nursing integration, and community health planning, creates a more explicit regulatory pathway for neighborhood-level access, but the Beijing case shows that this pathway remains contingent on financing, workforce capacity, facility readiness, and uneven implementation. The comparison therefore does not support a simple conclusion that one system is superior to the other. Rather, it shows that spatial accessibility depends on the interaction between land-use law, institutional design, social acceptance, and health-system capacity. The evidence presented here should therefore be interpreted as a structured legal-policy assessment rather than as a direct geospatial measurement of accessibility. The descriptive infrastructure indicators, policy timelines, and Seoul–Beijing case comparison do not measure patient travel time or facility catchment areas. Instead, they identify regulatory and institutional conditions that plausibly shape whether hospice services can be located close to residential communities.

The analysis yields three mechanism-level findings. First, clinical authorization and reimbursement policy do not by themselves create spatial accessibility unless they are matched by facility-siting pathways that allow hospice-compatible services to be located near residential communities. Second, community-embedded planning can create more favorable regulatory conditions for proximity, but only when supported by financing, workforce capacity, facility readiness, and quality governance. Third, NIMBYism should be understood not as an isolated cultural attitude, but as a governance problem shaped by facility classification, land cost, institutional design, and local implementation capacity.

These findings should not be read as suggesting that land-use regulation alone explains hospice accessibility. Health financing systems affect whether approved services can be sustained; workforce distribution affects whether formally accessible facilities can provide specialized palliative care; rural–urban disparities affect whether community-level infrastructure exists in the first place; and cultural attitudes toward death and dying affect whether legally permissible facilities are socially acceptable. Spatial regulation therefore operates within a broader health-system and cultural context, rather than as a single independent determinant.

Accordingly, the causal claims of this study are deliberately limited. The analysis does not claim that zoning rules alone cause inequitable hospice access, or that NIMBYism is the dominant determinant of facility distribution. Instead, it identifies a set of plausible regulatory and social mechanisms through which facility classification, siting permission, land acquisition, community delivery pathways, and social acceptance may condition the feasibility of community-adjacent hospice care.

Edward Soja’s spatial justice framework illuminates the distributive implications of these regulatory choices, highlighting how geographic distribution acts as a fundamental social determinant of health. Soja argued that justice has inherently geographic dimensions: where services are located determines who can access them, and spatial arrangements reflect and reproduce social inequities. Applied to hospice care, this framework helps interpret how seemingly neutral land-use regulations may create unequal conditions for accessing end-of-life care. Korean zoning rules that channel hospice-compatible facilities toward hospital, commercial, or peripheral locations may reduce the feasibility of receiving care in familiar community settings. Chinese planning standards that incorporate community health services within neighborhood-level service circles create a stronger formal basis for proximity, although this formal basis does not by itself guarantee actual service availability or quality. In this sense, spatial justice provides a normative framework for interpreting the distributive implications of facility-siting rules, rather than a direct empirical measure of accessibility (33, 34).

Henri Lefebvre’s concept of the “right to the city” extends this analysis to questions of participation and belonging, which are essential components for the psychosocial well-being of palliative patients. Lefebvre distinguished between mere “habitat”—reduced, functionalist space serving basic needs—and genuine “inhabiting”—full participation in the social life of urban space (35). For dying patients, this distinction is relevant because location can affect family presence, continuity of care, and perceived belonging. Peripheral or institutionally isolated hospice facilities may weaken these dimensions, while community-embedded services may better support care within familiar social environments. These claims should be understood as theoretical implications of the spatial justice framework, not as patient-level outcome findings from the present study. The spatial dimension of hospice care thus implicates not merely accessibility but the phenomenological quality of the dying experience.

The analysis reveals critical differences in land availability and social acceptance mechanisms across the two systems. Regarding land availability, Korea’s market-based system creates financial barriers that systematically disadvantage hospice facilities competing for urban land against commercial uses generating higher returns. China’s allocation system removes these financial barriers for qualifying institutions but introduces administrative discretion that may favor other priorities. The Korean approach produces predictable outcomes within constraining parameters; the Chinese approach offers flexibility that implementation may or may not realize.

Regarding social acceptance, both systems struggle with resident opposition to death-related facilities, but the mechanisms differ. Korean NIMBY conflicts typically occur at the project level, with residents opposing specific facility construction through protests, legal challenges, and political pressure. The documented cases—Inwae Dongsan, Daegu Buk-gu, Geumcheon Silber Center—illustrate this pattern of localized resistance. Existing research on the social acceptance of NIMBY facilities similarly suggests that resident concerns may be shaped by perceived risk, limited information, and distrust of siting procedures, while community-embedded service models may reduce the visibility of hospice care as a separate stigmatized facility.

Integration strategies may reduce some NIMBY pressures by expanding services within existing, accepted institutions, but they cannot eliminate cultural stigma or guarantee implementation quality.

The “aging in place” principle, endorsed by WHO’s Age-Friendly Cities framework (36), provides normative grounding for these spatial concerns. If older adults populations should be able to access necessary services within existing communities, this principle logically extends to end-of-life care. “Dying in place”—accessing hospice services without displacement from familiar surroundings—represents the natural culmination of aging in place (37). Yet both Korean and Chinese systems imperfectly realize this principle: Korean zoning often forces dying patients to relocate for inpatient hospice care, while Chinese community services may lack the capacity or quality to support complex end-of-life needs. Achieving genuine health equity in hospice care requires regulatory frameworks that both enable community-level service provision and ensure quality standards adequate to patient needs (38).

The following policy implications are derived from the six analytical indicators and the Seoul–Beijing case comparison, rather than from a general evaluation of national hospice policy. They should therefore be read as mechanism-specific recommendations addressing the bottlenecks identified above: facility classification, siting permission, land acquisition, community-embedded delivery, social acceptance, and implementation capacity. They also require attention to contextual modifiers—especially financing arrangements, workforce capacity, cultural acceptance, and rural–urban infrastructure disparities—because legal reforms in facility siting will have limited effect if these conditions remain unaddressed.

For this reason, the recommendations below are framed as staged implementation pathways rather than as immediately generalizable policy prescriptions. Their feasibility depends on political commitment from central and local governments, institutional coordination among planning, health, welfare, and insurance authorities, participation by hospice providers and community health institutions, and consultation with patients, families, residents, and local community organizations. The aim is not to suggest that spatial reform alone can resolve hospice accessibility, but to identify concrete entry points through which facility-siting reform can be coordinated with financing, workforce development, quality governance, and community acceptance.

Cross-national policy learning should therefore be selective and feasibility-oriented. Korea could consider targeted land-support mechanisms for hospice facilities, but such mechanisms would need to be adapted to Korea’s private land market, local zoning authority, and resident consultation procedures rather than borrowed wholesale from China’s administrative allocation model. Korea could also encourage limited hospice service integration within existing nursing hospitals and community health facilities, but only where reimbursement, workforce capacity, and quality standards are sufficient to support palliative care. Conversely, China could draw on Korea’s insurance coverage and designated-institution quality standards, but these mechanisms would need to be adapted to China’s fragmented reimbursement arrangements, regional disparities, and uneven community health infrastructure. Neither system therefore provides a complete model; each offers partial tools whose feasibility depends on local institutional capacity and stakeholder acceptance.

## 7 Conclusion

This comparative legal analysis has examined how health infrastructure planning frameworks shape the spatial distribution of hospice facilities in South Korea and China, two East Asian nations confronting rapid population aging with contrasting regulatory approaches. The investigation reveals that facility siting and geographic accessibility constitute a critical yet underrecognized bottleneck constraining hospice care accessibility—a hidden barrier operating beneath the surface of substantive hospice policy that has received increasing attention from lawmakers in both countries. While legislation governing hospice eligibility, clinical standards, and reimbursement has advanced significantly, the spatial dimension of end-of-life care remains subordinate to general medical facility zoning frameworks that were never designed to accommodate the distinctive needs of dying patients and their families.

In South Korea, the interaction of the National Land Planning and Utilization Act’s zoning categories, the Building Act’s facility classifications, and the Special Act on Development Restriction Zones produces a regulatory environment that channels hospice facilities away from residential communities. Standalone hospice facilities, classified as Category 9 medical facilities equivalent to hospitals, face the same zoning restrictions as general hospitals—permitted in Semi-Residential and Commercial zones, conditional in Natural Green Areas, and prohibited in Development Restriction Zones. This classification ignores the fundamental differences between acute care hospitals and hospice facilities, treating end-of-life care as merely another category of medical service rather than a distinct function requiring residential proximity. The documented NIMBY conflicts—from Inwae Dongsan’s criminal prosecutions to Daegu Buk-gu’s permit revocation—demonstrate that even where zoning technically permits development, social opposition can block facility construction.

In China, the Land Administration Law’s allocation provisions and the medical-nursing combined policy framework theoretically enable more flexible spatial distribution of hospice services. Non-profit medical and welfare facilities can receive land through administrative allocation without paying market-price land premiums, removing a significant financial barrier to hospice development. The 15-min Life Circle concept, codified in national urban planning standards, mandates community-level health and older adults care services within walking distance of residential areas, creating a framework for distributed hospice care access. However, implementation gaps, quality concerns, and regional disparities limit realization of these theoretical advantages. The 152 pilot regions represent substantial progress, but comprehensive national coverage remains distant, and the quality of hospice services within community-embedded facilities varies substantially.

The city-level cases of Seoul and Beijing concretize and qualify these conclusions. Seoul shows how medical-facility classification, greenbelt pressure, market-based land acquisition, and community resistance may jointly constrain the spatial embedding of end-of-life care even where substantive hospice policy has advanced. Beijing shows that community health planning and the 15-min life circle can create a formal pathway for neighborhood-level hospice access, but that this pathway remains limited by workforce capacity, reimbursement arrangements, facility readiness, and uneven district-level implementation. These cases support a moderated conclusion: spatial planning does not mechanically determine hospice accessibility, but it creates the regulatory and institutional conditions under which access becomes more or less feasible.

The health equity implications of these findings extend beyond the specific cases of South Korea and China, highlighting geographic accessibility as a universal social determinant of health. Edward Soja’s framework reminds us that justice has inherently geographic dimensions—that where services are located determines who can access them, and that spatial arrangements both reflect and reproduce social inequities. The pattern documented in this study, where regulatory frameworks designed for general purposes produce systematically unequal access to end-of-life care, likely characterizes other aging societies grappling with hospice facility siting. European nations facing similar demographic transitions, including Germany, Italy, and Spain, may discover comparable tensions between existing land use frameworks and emerging hospice care needs. The NIMBY dynamics documented in Korean cases find echoes in facility siting conflicts throughout the developed world.

Based on this analysis, we offer the following policy recommendations.

First, for South Korea, a distinct facility classification for small-scale hospice facilities could be developed through a phased pilot rather than immediate nationwide reform. The Ministry of Land, Infrastructure and Transport, the Ministry of Health and Welfare, municipal governments, hospice providers, and resident representatives could first identify a limited category of small hospice facilities, for example facilities below a specified bed threshold, subject to design, staffing, noise, traffic, infection-control, and family-visitation standards. Pilot authorization in selected residential-adjacent or mixed-use zones would allow policymakers to assess whether such facilities can operate compatibly with neighborhood life before broader amendment of the Building Act or zoning rules. This pathway would address the Korean classification problem while acknowledging political resistance, municipal discretion, and resident concerns.

Second, for both countries, we recommend mandatory inclusion of hospice care capacity within urban renewal and new development frameworks (39, 40). Just as many jurisdictions require affordable housing set-asides in new residential developments, planning regulations could require allocation of space for end-of-life care services within urban regeneration projects (41). This approach would address systemic health inequity concerns by ensuring that hospice care capacity expands in tandem with residential development rather than lagging behind demographic change. The integration could take various forms: dedicated hospice units within mixed-use developments, reserved space for hospice services within community health facilities, or land parcels designated for future hospice use within new residential districts.

Third, compensation and consultation mechanisms should be designed as part of the siting process rather than added only after conflict emerges. Structured community benefit agreements could provide tangible local benefits, such as improvements to community facilities, traffic management, green space, neighborhood health services, or public information programs. More importantly, these agreements should be negotiated through transparent procedures involving local residents, municipal governments, facility operators, health authorities, and community organizations. Such mechanisms would not eliminate opposition, but they could reduce distrust, clarify expected neighborhood impacts, and create institutional channels for mediation before disputes escalate into litigation or permit revocation.

Fourth, both countries should invest in public education campaigns addressing the cultural stigma surrounding death and hospice care. The NIMBY research consistently identifies superstitious beliefs and limited knowledge as factors driving opposition to death-related facilities. Changing these attitudes requires sustained engagement with communities, involving both information dissemination about the nature of hospice services and normative messaging about dignified dying as a community concern rather than a private matter to be hidden from view. Media representations of hospice care, engagement with religious and cultural leaders, and incorporation of end-of-life care topics in health education curricula could contribute to gradual attitude shifts enabling greater social acceptance of proximate hospice facilities (42).

This study has several limitations that should guide interpretation of findings and future research. First, the analysis relies exclusively on legal texts, policy documents, and secondary sources; no primary data collection through surveys, interviews, or site visits was conducted. This approach was appropriate for comparative legal analysis but limits our ability to assess implementation dynamics that may diverge significantly from policy intent. Consequently, the perspectives of patients, family caregivers, hospice professionals, facility operators, local residents, urban planners, and policymakers are not directly captured. The policy implications should therefore be interpreted as legal-policy proposals derived from document analysis and illustrative cases, rather than as stakeholder-validated implementation plans. Second, the NIMBY case documentation is illustrative and case-based, and the documentation for South Korea is more extensive than for China, potentially creating asymmetric evidence bases for the two countries. These cases help show how social resistance may interact with facility classification, land cost, and local governance, but they cannot establish the prevalence, causal weight, or independent effect of NIMBYism across all hospice-related facility siting decisions. Third, the study focuses on formal regulatory frameworks without systematic analysis of informal practices that may substantially affect actual facility siting outcomes. Fourth, health financing systems, cultural attitudes toward death and dying, rural–urban disparities, and healthcare workforce distribution are considered qualitatively as contextual modifiers rather than modeled as independent causal variables. This approach helps avoid a single-factor explanation of hospice accessibility, but it cannot isolate the relative weight of each contextual factor or determine how these factors interact across different regions and facility types. Fifth, this study does not provide a full GIS-based accessibility analysis, patient-level travel-time measurement, or quantitative modeling of hospice facility distribution. Geographic accessibility is therefore assessed through legal-policy proxy indicators rather than direct spatial measurement. This approach is useful for identifying regulatory and institutional conditions that shape siting feasibility, but it cannot determine actual travel distance, catchment-area coverage, or patient-level access. The Seoul and Beijing cases should also be understood as illustrative case studies rather than statistically representative evidence. They were selected to examine how national regulatory frameworks are translated into concrete metropolitan contexts, not to establish generalizable causal effects across all cities in South Korea and China. The cases provide contextual support for the comparative analysis by linking national policy frameworks to city-level implementation dynamics, but they should be interpreted as complementary qualitative evidence rather than as substitutes for broader empirical validation.

Future research should address these limitations through empirical investigation of hospice facility spatial distribution patterns, including GIS-based accessibility analysis, patient-level data on distance to care, and comparable data on reimbursement, workforce distribution, rural–urban infrastructure capacity, and public attitudes toward end-of-life care. It should also incorporate stakeholder interviews or surveys with patients, family caregivers, hospice professionals, facility operators, residents, urban planners, and policymakers to evaluate the feasibility, acceptability, and implementation risks of the policy pathways proposed in this study. Qualitative research exploring the decision-making processes of hospice facility developers, including their navigation of regulatory barriers and NIMBY opposition, would illuminate dynamics invisible in legal text analysis. Comparative research extending to additional East Asian jurisdictions—particularly Japan and Taiwan, which share cultural context but have developed distinct regulatory approaches—would strengthen the evidence base for cross-national policy learning. Research specifically examining the quality implications of community-embedded hospice services versus specialized standalone facilities would inform the integration-versus-specialization debate with outcome data rather than theoretical speculation.

In conclusion, this study suggests that achieving health equity in hospice care requires greater attention to structural infrastructure planning frameworks that have received limited scrutiny in end-of-life care policy discourse. Its main contribution is to show, through a structured comparison of legal-policy indicators and two city-level cases, how facility classification, land acquisition, community delivery pathways, social acceptance, and implementation capacity jointly shape the regulatory conditions for hospice accessibility. The “spaces of dying” are not merely medical or social phenomena but spatial phenomena shaped by regulatory choices about where facilities may be built and how competing land uses are balanced (43). As East Asia’s Silver Tsunami continues to reshape demographic realities, the urgency of addressing these spatial dimensions will only intensify. The policy recommendations offered here should therefore be understood as starting points for a broader discussion about how end-of-life care can be integrated into community planning while remaining attentive to financing, workforce, quality, rural–urban inequality, and social acceptance constraints. In normative terms, the analysis supports the proposition that dying, like living, deserves a place in the city (44).

## Data Availability

Publicly available datasets were analyzed in this study. This data can be found here: official government databases including the Korea Ministry of Government Legislation (MOLEG), China’s National People’s Congress (NPC) database, Statistics Korea (KOSIS), and China’s National Bureau of Statistics.
