# Supplementary material for: Navigating spatial barriers in healthcare: health equity and end-of-life care infrastructure in South Korea and China
Source: Front Public Health. 2026 Jul 13;14:1827548. doi: 10.3389/fpubh.2026.1827548 (PMC13402389; doi:10.3389/fpubh.2026.1827548)
Supplement: Supplementary file 1 [file Table_1.DOCX]

**Supplementary Table S1. Expanded city-level analytical matrix for Seoul and Beijing**

| **Analytical dimension** | **Seoul / Seoul Metropolitan Area** | **Beijing** | **Implication for spatial accessibility analysis** |
| --- | --- | --- | --- |
| Case selection rationale | Seoul represents a dense Korean metropolitan context where land scarcity, high land prices, zoning restrictions, greenbelt pressure, and local opposition converge. | Beijing represents a Chinese metropolitan context where community health planning, medical-nursing integration, and the 15-minute life circle are formally incorporated into urban service provision. | The two cases allow national legal and policy frameworks to be examined in concrete metropolitan settings. |
| Urban spatial context | High-density urban development and intense competition for land make community-adjacent siting difficult for welfare and medical facilities. | Large-scale urban governance and hierarchical planning create opportunities to integrate health and older-adult care services into community infrastructure. | Metropolitan land pressure affects both cities, but each system responds through different institutional tools. |
| Land ownership and land acquisition | Facility developers generally rely on market-based land acquisition, which increases costs and disadvantages low-profit welfare or hospice-related facilities. | Public land allocation and administrative planning can support public welfare and community health facilities, although local discretion remains important. | Land acquisition mechanisms influence whether hospice-compatible services can be embedded close to residents. |
| Land-use and zoning framework | Hospice care is shaped by general medical-facility zoning rules, hospital classifications, and restrictions in residential or greenbelt-adjacent areas. | Community health and older-adult care services can be planned through public-service facility standards and neighborhood service infrastructure. | Zoning-based exclusion and planning-based embedding represent two different approaches to spatial governance. |
| Facility classification | Hospice care is generally not treated as a distinct land-use category. Hospice units inherit the classification of hospitals or medical facilities. | Hospice-compatible care may be incorporated into community health service centers, nursing care institutions, or medical-nursing integrated facilities. | Classification determines whether hospice care is viewed as a specialized institution or as a function within existing community services. |
| Community-level access pathway | Access is mainly mediated through hospitals, nursing hospitals, or formally approved medical facilities. This may limit small-scale, family-oriented, community-adjacent models. | Community health service centers provide a possible platform for neighborhood-level hospice-compatible services. | Community access depends not only on whether hospice care is legally authorized, but also on the institutional form through which it is delivered. |
| Concrete city-level evidence | The Geumcheon Silber Center dispute illustrates how older-adult care facilities can become targets of neighborhood opposition. Seoul’s broader debates over non-preferred facilities also show the political sensitivity of death-related or welfare-related siting. | Beijing’s community health service center standards illustrate a more concrete pathway for integrating rehabilitation, nursing, and hospice-compatible beds into neighborhood health infrastructure. | The cases provide contextual support for the claim that national rules operate through local land-use, institutional, and social dynamics. |
| Social acceptance and NIMBY dynamics | Local resistance may arise from concerns over stigma, property values, traffic, emotional discomfort, and distrust of government-led siting decisions. | Resistance may be less visible when hospice-compatible services are embedded in accepted community health institutions rather than developed as separate death-associated facilities. | NIMBYism should be understood as interacting with facility design, legal classification, and institutional embedding, rather than as an isolated cultural attitude. |
| Cultural framing of death-related services | Death-adjacent services may be associated with misfortune, stigma, or unwanted neighborhood identity, increasing the political cost of siting. | Similar cultural concerns may exist, but embedding services within broader health and aging-care institutions may dilute the visibility of death-related functions. | Cultural stigma affects both cities, but institutional design may shape how strongly it appears in siting conflicts. |
| Financing and reimbursement | South Korea has a more developed hospice reimbursement framework, but payment policy does not automatically overcome spatial siting barriers. | China’s hospice reimbursement remains more fragmented across regions and schemes, which may weaken the practical implementation of community-based services. | Financing operates as a contextual modifier: it can support service sustainability but cannot by itself resolve spatial barriers. |
| Workforce and service quality | Korea’s designated hospice institutions may offer more standardized clinical quality, but accessibility can remain constrained by location. | Beijing’s community-embedded pathway may improve proximity, but service quality depends on trained personnel, facility readiness, and palliative care capacity. | The access-quality tradeoff is central: spatial proximity must be paired with adequate professional capacity. |
| District-level or regional inequality | In Seoul, unequal land availability and neighborhood resistance may affect where facilities can realistically be located. | In Beijing, implementation may vary across central urban districts, suburban areas, and mountainous areas, despite formal accessibility standards. | City-level planning standards do not automatically guarantee equal access across all districts. |
| Relation to national-level findings | Seoul concretizes the Korean pattern: clinical and insurance policy development may coexist with unresolved spatial barriers. | Beijing concretizes the Chinese pattern: planning integration creates a formal access pathway but remains limited by implementation capacity. | The cases support the national comparison while moderating overly broad causal claims. |
| Main limitation of the case evidence | The Seoul case is illustrative and cannot prove that zoning alone causes hospice inaccessibility across all Korean cities. | The Beijing case is illustrative and cannot prove that community planning produces actual hospice access across all Chinese cities. | The cases provide contextual validation rather than statistical generalization. |
| Policy implication | Seoul suggests the need for more flexible facility classification, zoning exceptions for small-scale hospice care, resident consultation, and compensation or mediation mechanisms. | Beijing suggests the need to link community planning standards with reimbursement, workforce training, quality control, and district-level monitoring. | Effective hospice accessibility policy requires integrating land-use planning with health financing, workforce development, and community governance. |
